# Perfluoroalkyl substances: a risk for the aquatifc environment? A 1-year case study in river waters of central Italy

**DOI:** 10.1007/s11356-024-34807-4

**Published:** 2024-09-18

**Authors:** Federica Castellani, Mara Galletti, Fedra Charavgis, Alessandra Cingolani, Sonia Renzi, Mirko Nucci, Carmela Protano, Matteo Vitali

**Affiliations:** 1 Department of Public Health and Infectious Diseases, University of Rome La Sapienza, P.le Aldo Moro, 5, Rome, 00185 Italy; 2 ARPA Umbria, Via Carlo Alberto Dalla Chiesa, 23, 05100 Terni, Italy

**Keywords:** PFASs, River water, River pollution, POPs, ERA, Risk quotient

## Abstract

Perfluoroalkyl substances (PFASs) are a large class of persistent emerging pollutants, ubiquitous in different environmental compartments. In this study, twenty-one PFASs were determined in seventy-eight water samples collected from six different rivers in the Umbria region (central Italy) during a 13-month monitoring campaign. The sum of the twenty-one target analytes (Σ_21_PFASs) ranged from 2.0 to 68.5 ng L^−1^, with a mean value of 22.0 ng L^−1^. The highest concentrations of Σ_21_PFASs were recorded in the warmest months (from June to September) due to reduced river streamflow caused by low rainfall and high temperatures. PFASs with a number of carbon atoms between four and nine prevail over C10–C18 congeners due to their higher water solubility and to their increased use in industry. PFBA, followed by PFPeA, PFHxA, and PFOA, was the most abundant congeners detected in the analyzed river water samples. Finally, the calculation of risk quotients (∑RQs) has allowed to assess the risk for three aquatic organisms (fish, algae, and daphnid) deriving from the exposure to PFASs. The survey showed that the risk for the three aquatic organisms during the four seasons and throughout the year was always negligible. The only exception was a low risk for fish and daphnid in GEN river considering the annual exposure.

**Supplementary Information:**

The online version contains supplementary material available at 10.1007/s11356-024-34807-4.

## Introduction

Perfluoroalkyl substances (PFASs) are a large group of synthetic organic chemicals widely utilized in everyday products, such as food packaging, kitchenware, fabric, coatings, and electronics (Cai et al. 2018). PFASs are characterized by a fully fluorinated carbon chain and a hydrophilic head group (a carboxylic or sulphonic acid); this peculiar chemical structure gives the substances amphiphilic properties, widely exploited in the industrial field since 1960s (Zhang et al. 2021a, b). In addition, the strength of carbon-fluoride (C-F) bond makes PFASs extremely resistant to any degradation process, such as biodegradation, photolysis, hydrolysis, and also metabolism (Organisation for Economic Co-operation and Development 2018; Castellani et al. 2023). Among more than 3000 perfluoroalkyl congeners, PFASs with a number of carbon atoms ≥ 6 cause great concern for human health due to their toxicity and bioaccumulation potential (Leng et al. 2021). Since 2009, in fact, perfluorooctane sulfonic acid (PFOS, 8 carbon atoms) and its salts were listed in the annex B of the Stockholm convention (UNEP 2009) to restrict their production and use. Ten years later, also perfluorooctanoic acid (PFOA, eight carbon atoms) and its salts were listed in the annex A of the Stockholm convention (UNEP 2019) to eliminate their production and use. In 2022, perfluorohexane sulfonic acid (PFHxS, six carbon atoms) and its salts were listed in the annex A of the Stockholm convention (decision SC-10/13; UNEP 2022a) and long-chain perfluorocarboxylic acids (LC-PFCAs, number of carbon atoms between nine and twenty-one) and their salts were proposed for listing in annexes A, B, or C of the Stockholm Convention (UNEP 2022b). The different length of the carbon chain implies different behavior in term of migration, degradation, and bioaccumulation of PFASs. Specifically, short-chain PFASs are more persistent and mobile in water media in comparison to long-chain PFASs that tend to accumulate in sediments (Chen et al. 2019). Long and short-chain PFASs also differ regarding the elimination rate from human body: short-chain compounds were excreted much more quickly than long-chain PFASs (Yao et al. 2018).

Despite short-chain PFASs are more subject to long-range transport and as persistent as long-chain PFASs (Yao et al. 2018; Li et al. 2020a, b; Zhang et al. 2021a, b), they are not yet regulated because of their lower toxicity and lower bioaccumulation potential (Wu et al. 2020; Zhang et al. 2022). This regulatory revolution has been implemented following the contamination of several environmental compartments, as well as living organisms, by PFASs (Zafeiraki et al. 2019; Zhou et al. 2021; Nayak et al. 2023). Due to the low volatility and high hydrophilicity characterizing this class of compounds, PFASs are mainly detected in aquatic environments (Wang et al. 2022). The contamination sources of aquatic matrices can be both direct (such as wastewater treatment plants or industrial discharges) or indirect (such as long-range transport or transformation of chemical precursors) (Dasu et al. 2022; Saawarn et al. 2022). It is well known that the exposure of aquatic organisms to PFASs can produce multiple toxic effects including lipid and carbohydrate metabolism alteration, oxidative stress, endocrine and thyroid disruption, apoptosis, reproductive, neurodevelopmental and immune toxicity, and growth inhibition (Lee et al. 2020; Mahoney et al. 2022). PFASs can also exert toxic effects on algae by reducing photosynthetic efficiency, by causing the accumulation of reactive oxygen species (ROS) that induce oxidative stress and by interrupting DNA replication, inhibiting algae growth (Liu et al. 2022).

In this study, the presence and seasonal trend of twenty-one PFASs detected in six different rivers of Umbria region (central Italy) were investigated during a 13-month monitoring campaign. The collected contamination data were then used to track the possible emission sources of PFASs detected in river water samples. Finally, the potential risks for the aquatic flora and fauna (algae, fish, and daphnid) deriving from the exposure to PFASs were assessed through the calculation of risk quotients (RQs). This is, to our knowledge, the first study that investigates the presence and seasonal trend of PFASs in river water samples of central Italy. Additionally, the ecological risk assessment was a useful tool to evaluate the health status of the studied rivers.

## Materials and methods

### Sample collection

**Fig. 1:**
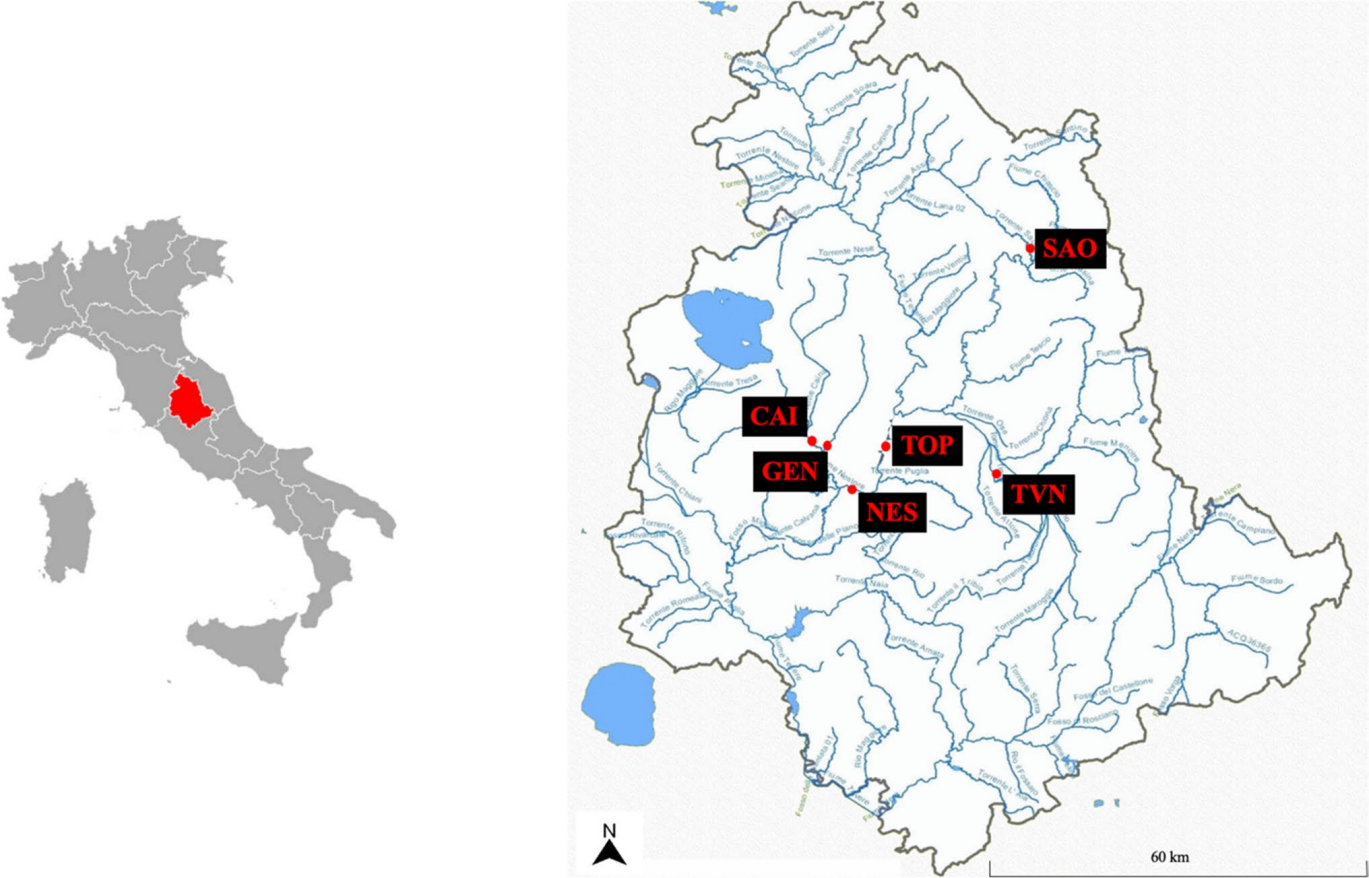
Map of the six rivers (CAI, GEN, TOP, NES, TVN, and SAO) in Umbria region (central Italy)

Seventy-eight river water samples were collected from six different rivers of Umbria region (central Italy; Fig. 1) during a 13-month monitoring campaign (March 2022–March 2023).

The rivers were selected based on a previous study conducted by the regional agency for environmental protection of Umbria (Nucci et al. 2019a,b; Charavgis et al. 2022), in which emerged that these rivers exceeded the maximum levels of PFASs fixed by directive 2013/39/EU (European Commission 2013). Additionally, the six selected rivers were affected by potential sources of contamination as reported in Table 1. All the river water samples were collected monthly, using 0.5 L polypropylene (PP) bottle, pre-cleaned with methanol and ultrapure water. Due to the surfactant properties of PFASs, river waters were collected as grab samples, as indicated by EPA (Environmental Protection Agency; EPA 2022). The samples were refrigerated, transported to the laboratory, and stored at + 4 °C until analysis. Sampling details and area description are reported in Table 1.

**Table 1:** Sampling specifications, area description, and main sources of contamination affecting the studied rivers

River	Geographic coordinates	Area description	Main sources of contamination
CAI	12° 15′ 44.73″ E 43° 0′ 9.06″ N	Rural area	Water plants, urban wastewater, industrial facilities, agricultural and livestock
NES	12° 21′ 58.61″ E 42° 54′ 27.60″ N	Urban/industrial area	Wastewater plants, urban wastewater, industrial facilities, agricultural and livestock
GEN	12° 17′ 29.24″ E 42° 58′ 8.93″ N	Rural area	Wastewater plants, urban wastewater, industrial facilities, agricultural and livestock
TOP	12° 30′ 27.33″ E 43° 1′ 34.51″ N	Urban/rural area	Wastewater plants, urban wastewater, industrial facilities, agricultural and livestock
SAO	12° 39′ 25.20″ E 43° 15′ 45.79″ N	Rural area	Urban wastewater, industrial facilities, agricultural and livestock
TVN	12° 36′ 38.53″ E 42° 55′ 50.37″ N	Urban/industrial area	Wastewater plants, urban wastewater, agricultural and livestock

### Chemicals and reagents

LC–MS grade methanol (MeOH) was purchased by Merck (Darmstadt, Germany), and ultrapure water was obtained from a Milli-Q filter system (Millipore, Bedford, MA, USA). HPLC grade ammonium acetate was supplied by Merck (Darmstadt, Germany). Stock standard solutions containing 2 μg mL^−1^ of the target analytes (PFBA, PFPeA, PFBS, PFHxA, PFPeS, PFHpA, PFHxS, PFOA, PFHpS, PFNA, PFOS, PFDA, PFNS, PFUdA, PFDS, PFDoA, PFTriA, PFDoS, PFTeA, PFHxDA, and PFODA) were obtained from Wellington Laboratories Inc. (Guelph, Ontario, Canada). Mass-labeled injection standards (IS; M3PFBA, M2PFOA, MPFOS, and MPFDA) and mass-labeled extraction standards (ES; MPFBA, M5PFPeA, M3PFBS, M5PFHxA, M4PFHpA, M3PFHxS, M8PFOA, M9PFNA, M8PFOS, M6PFDA, M7PFUdA, MPFDoA, and M2PFTeDA) at a concentration of 2 μg mL^−1^ were purchased from Wellington Laboratories Inc. (Guelph, Ontario, Canada). Full names and internal standards of the target chemicals are listed in supplementary material in Table S1.

### Sample extraction and instrumental analysis

The sample extraction and purification were performed following EPA method 533 (US EPA, 2019), as extensively described in Castellani et al. (2023). Briefly, 250 mL of river water samples, previously spiked with 250 μL of the ES at the concentration of 20 ng mL^−1^, were loaded onto Strata™-XL-AW cartridge (100 mg, 6 mL, Phenomenex, CA, United States). Target PFASs were eluted into a polypropylene tube using 10 mL of MeOH containing 2% of NH_4_OH. The samples were then dried under a gentle nitrogen flux and then re-suspended in 250 μL of IS solution (20 ng mL^−1^). Chromatographic separation was performed by HPLC Agilent 1290 Infinity II (Agilent Technologies, Santa Clara, CA, USA) equipped with Zorbax Eclipse Plus C18 RRHD (50 × 3.0 mm, 1.8 μm) chromatographic column purchased from Agilent Technologies (Santa Clara, CA). A delay column (Zorbax Eclipse Plus C18 RRHD, 4.6 × 30 mm, Agilent Technologies, USA) was installed between the solvent mixer and injector module to avoid instrumental contamination. Chromatography was performed using H_2_O + 2 mM of ammonium acetate (A) and MeOH + 2 mM of ammonium acetate (B) at a flow rate of 0.5 mL min^−1^. Gradient elution started at 40% of B for 0.5 min and was raised to 80% within 7.5 min; after 4 min in isocratic condition, B was raised to 95% and equilibrated for 1 min. The initial conditions were then restored, and the system was equilibrated for 2 min. The column temperature was 40 °C, and the injection volume was 5 μL. The retention times of the target analytes are reported in Table 2. The HPLC was interfaced to an Agilent 6475 triple quadrupole mass spectrometer with a Jet Stream 6450 electrospray ionization unit (AJS-ESI) operating in multiple reaction monitoring (MRM) negative detection mode. The source and sheath gas temperatures were set at 320 °C and 350 °C, respectively. The nozzle voltage and the ion capillary were 1500 V and 3750 V, respectively. The gas and the sheath gas flow were set at 5 L min^−1^ and 12 L min^−1^. For all the analytes under study, the nebulizer was 50 psi and the cell accelerator voltage was 7 V. The collision energies and the fragmentor values set for the target analytes were reported in Table 2, together with precursor and product ions chosen for the quantification.

**Table 2:** Full names, acronym, internal standards, retention times (RT; minutes), precursor and product ions (q: qualifier, Q: quantifier), fragmentor (V), and collision energy (eV) of the target analytes

Full name	Acronym	Internal standard	RT (min)	Precursor ion (m/z)		Product ion (m/z)	Fragmentor (V)	Collision energy (eV)
Perfluorobutanoic acid	**PFBA**	M3PFBA	0.81	213		169	60	8
Perfluoropentanoic acid	**PFPeA**	M5PFPeA	1.98	263		219		6
Potassium perfluoro-1-butanesulfonate	**PFBS**	M3PFBS	2.08	298.9	Q	80	133	45
					q	98.9		29
Perfluorohexanoic acid	**PFHxA**	M5PFHxA	3.56	312.9		268.9	66	5
Sodium perfluoro-1-pentanesulfonate	**PFPeS**	M3PFHxS	3.82	349	Q	80	135	40
					q	99		36
Perfluoroheptanoic acid	**PFHpA**	M4PFHpA	5.00	362.9	Q	319	66	5
					q	169		13
Sodium perfluoro-1-hexanesulfonate	**PFHxS**	M3PFHxS	5.13	398.9	Q	80	174	49
					q	99		45
Perfluorooctanoic acid	**PFOA**	M8PFOA	6.10	412.9	Q	368.9	86	5
					q	169		13
Sodium perfluoro-1-heptanesulfonate	**PFHpS**	M8PFOS	6.17	449	Q	80	100	50
					q	99		46
Perfluorononanoic acid	**PFNA**	M9PFNA	7.09	462.9	Q	418.9	66	5
					q	169		17
Sodium perfluoro-1-octanesulfonate	**PFOS**	M8PFOS	7.09	498.9	Q	80	210	50
					q	99		50
Perfluorodecanoic acid	**PFDA**	M6PFDA	7.74	512.9	Q	469	102	5
					q	169		20
Sodium perfluoro-1-nonanesulfonate	**PFNS**	M8PFOS	7.65	549	Q	80	165	76
					q	99		48
Perfluoroundecanoic acid	**PFUdA**	M7PFUdA	8.45	562.9	Q	519	92	5
					q	169		21
Sodium perfluoro-1-decanesulfonate	**PFDS**	M8PFOS	8.38	598.9	Q	80	120	94
					q	99		60
Perfluorododecanoic acid	**PFDoA**	MPFDoA	9.04	612.9	Q	569	97	5
					q	169		25
Perfluorotridecanoic acid	**PFTriA**	M2PFTeDA	9.56	662.9	Q	619	102	9
					q	169		30
Sodium perfluoro-1-dodecanesulfonate	**PFDoS**	M8PFOS	9.42	699	Q	80	100	64
					q	99		60
Perfluorotetradecanoic acid	**PFTeA**	M2PFTeDA	10.08	712.9	Q	669	112	9
					q	169		40
Perfluorohexadecanoic acid	**PFHxDA**	M2PFTeDA	11.92	813	Q	769	100	15
					q	169		40
Perfluorooctadecanoic acid	**PFODA**	M2PFTeDA	13.38	913	Q	869	200	15
					q	169		40

### Statistical analysis

Principal component analysis (PCA) was performed by using R software (R-project for statistical computing, version 3.0, 32-bit). PCA was carried out with the aim of clustering the possible tracers of the main emission sources affecting the six different rivers under study. Column mean centering and row and column autoscaling were applied before performing PCA to correct the matrix of the data for different variable scaling and units.

### Ecological risk assessment

To assess the risk for the aquatic biota (fish, algae, and daphnid), the risk quotients (RQs) were calculated, as suggested by Leng et al. (2021), following Eqs. (1) to (3) for sixteen of twenty-one PFASs under study. The choice of the compounds was based on the different solubility of PFASs in the water matrix; for this reason, C-12 sulphonic acid and C-13,14,18 carboxylic acids were excluded from the RQ calculation. The ∑RQs were calculated both seasonally and annually for all the six rivers under study. As reported by Li et al. (2020a), the value of the calculated RQ provides information on the different risk categories: RQ < 0.01: negligible risk, 0.01 < RQ < 0.1: low risk, 0.1 < RQ < 1: medium risk, and RQ > 1: high risk.

1 $$\mathrm{RQ}=\frac{\mathrm{MEC}}{\mathrm{PNEC}}$$

2 $$\mathrm{PNEC}=\frac{{\mathrm{LC}}_{50}\;{\mathrm{or\;EC}}_{50}}{\mathrm{AF}}$$

3 $${\mathrm{RQ}}_{\mathrm{total}}=\sum {\mathrm{RQ}}_{i}$$

where MEC is the measured environmental concentration (ng L^−1^) expressed utilizing the upper bound approach (worst case scenario: values < LOD are equal to LOD value), PNEC is the predicted no-effect concentration (ng L^−1^), and LC_50_ and EC_50_ are the median lethal concentration and the median effective concentration, respectively. The toxicity data of PFASs (LC_50_ or EC_50_) were estimated using EPIWEB 4.1, and the details were reported in Table 3. Finally, AF is the assessment factor, which is set at 100 for chronic toxicity.

**Table 3:** Toxicity data (LC_50_ or EC_50_; ng L^−1^) of PFASs for three aquatic organisms (fish, algae, and daphnid)

	Fish	Algae	Daphnid
PFBA	1.32E + 09	5.97E + 08	7.61E + 08
PFPeA	4.09E + 08	2.54E + 08	2.50E + 08
PFBS	3.60E + 09	1.40E + 09	2.01E + 09
PFHxA	1.22E + 08	1.04E + 08	7.93E + 07
PFPeS	1.05E + 09	5.60E + 08	6.25E + 08
PFHpA	3.55E + 07	4.14E + 07	2.45E + 07
PFHxS	3.01E + 08	2.20E + 08	1.90E + 08
PFOA	1.01E + 07	1.62E + 07	7.44E + 06
PFHpS	8.50E + 07	8.54E + 07	5.71E + 07
PFNA	2.84E + 06	6.26E + 06	2.22E + 06
PFOS	2.37E + 07	3.27E + 07	1.69E + 07
PFNS	6.53E + 06	1.24E + 07	4.96E + 06
PFDA	7.90E + 05	2.39E + 06	6.60E + 05
PFDS	1.78E + 06	4.64E + 06	1.44E + 06
PFUdA	2.20E + 05	9.00E + 05	1.90E + 05
PFDoA	5.90E + 04	3.40E + 05	6.00E + 04

## Results and discussion

### PFAS concentrations and monthly variations

**Fig. 2:**
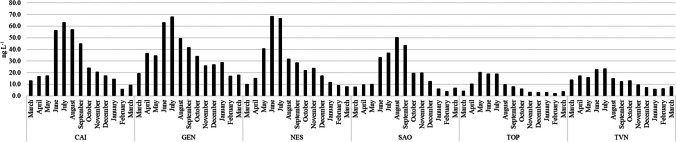
Monthly trend (March 2022–March 2023) of the sum of twenty-one PFASs detected in the selected river water samples (CAI, GEN, NES, SAO, TOP, and TVN) in Umbria region (central Italy)

The results of the analysis of river water samples (*n* = 1638 analytical determinations) are summarized in Table 4. Among twenty-one target compounds, only the long-chain PFASs (PFNS, PFDS, PFUdA, PFDoA, PFDoS, PFTrDA, PFTeA, PFHxDA, and PFODA) were detected in less than 50% of the analyzed river water samples. The concentrations of PFBA, PFBS, PFPeA, PFHxA, and PFOA were below the regulatory limits fixed by Italian 172/2015 Decree Law (Environmental Quality Standard (EQS): 7000 ng L^−1^ for PFBA, 3000 ng L^−1^ for PFBS and PFPeA, 1000 ng L^−1^ for PFHxA, and 100 ng L^−1^ for PFOA) in all the sampling months (Table 4). The concentration of PFOS, however, exceeded the maximum level fixed by Italian 172/2015 Decree Law (0.65 ng L^−1^) in 47% of the analyzed samples (Table 4). PFOS concentrations ranged from < LOQ to 2.2 ng L^−1^, with a mean of 0.8 ng L^−1^; in detail, the highest concentrations of PFOS were recorded in the warmest month (from June to September) in all the rivers under study. GEN was the only river in which PFOS concentrations exceeded the fixed EQS in all the sampling months. On the other hand, TOP river was the only river in which PFOS did not exceed the fixed EQS in any sampling month. Castiglioni et al. (2015) measured PFOS concentration in five different rivers in northern Italy, finding values between < LOQ and 43 ng L^−1^, with a mean value of 14 ng L^−1^. These values are 18 times higher than those reported in this study (Table 4). Valsecchi et al. (2015) measured PFOS concentration in waters collected from five different rivers in the most industrialized area of northern Italy, finding concentrations between < LOD and 150 ng L^−1^. Also, in this case, the concentrations detected are much higher than those measured in this work. More recently, Barreca et al. (2020) reported PFOS concentrations between < LOQ and 29.4 ng L^−1^ in 57 river water samples collected in northern Italy, values 13 times higher than those reported in this study (Table 4). Llorens et al. (2020) measured PFOS in sixteen different river waters collected in northeastern Spain, finding concentrations between < LOD and 1.5 ng L^−1^ with a mean concentration of 0.039 ng L^−1^. These values are lower than PFOS concentrations reported in this work (Table 4). Regarding the congeners distribution, PFASs with a number of carbon atoms between four and nine prevail over the long-chain congeners (C10–C18; Table 4). This trend could be due to the higher water solubility of short-chain PFASs and to the industrial replacement of long-chain congeners on behalf of the short ones. Also, Selvaraj et al. (2021) found a similar trend in Indian rivers. PFBA, followed by PFPeA, PFHxA, and PFOA, was the most abundant congeners detected in river water samples (Table 4). Giglioli et al. (2023) reported PFBA, followed by PFOA and PFBS, as major congeners detected in superficial waters of northern Italy. The sum of 21 target PFASs (∑_21_PFASs) detected in the six selected river water samples ranged from 2.0 to 68.5 ng L^−1^ (Fig. 2), with a mean value of 22.0 ng L^−1^. As shown in Fig. 2, the ∑_21_PFASs were higher in the warmest months (from May to August) in all the rivers under study. The ∑_21_PFASs were higher in NES river (68.5 ng L^−1^), followed by GEN and CAI rivers (67.9 and 62.9 ng L^−1^, respectively). SAO, TVN, and TOP were the rivers with the lowest ∑_21_PFASs (50.2, 23.3, and 20.4 ng L^−1^, respectively; Fig. 2). Zhu et al. (2015) measured ∑PFAS concentration in Daling River (northeast China) in different seasons recording concentrations between 1.77 and 9540 ng L^−1^, with a seasonal trend characterized by higher concentrations in warmer months. Although the seasonal trend is the same, the concentrations of ∑PFASs recorded by Zhu et al. (2015) are significantly higher than those reported in this study (from 2.0 to 68.5 ng L^−1^, Fig. 2). Munoz et al. (2019) evaluated the temporal variation of PFASs during 1-year monitoring campaign of the Gironde River (South-West France), finding ∑PFAS values between 3.5 and 11 ng L^−1^, with a median value of 6.2 ng L^−1^. These values are lower than those reported in this study (values between 2.0 and 68.5 ng L^−1^, with a median value of 17.1 ng L^−1^). The temporal trend reported by Munoz et al (2019) showed higher concentrations of ∑PFASs in the coldest month (November–January) compared to the warmest months (May–July). This trend is not in agreement with those reported in this study, in which the ∑PFASs are higher in the warmer month (Fig. 2), probably due to a reduced river flow caused by high temperatures and a lack of rain.

**Table 4:** Concentrations (ng L^−1^) of twenty-one PFASs detected in six river water samples in Umbria region (central Italy)

		PFBA	PFPeA	PFBS	PFHxA	PFPeS	PFHpA	PFHxS	PFOA	PFHpS	PFNA	PFOS	PFNS	PFDA	PFDS	PFUdA	PFDoA	PFDoS	PFTrDA	PFTeA	PFHxDA	PFODA
CAI	**March**	2.25	2.50	0.49	2.18	0.08	0.81	0.44	1.97	0.04	0.39	0.79	0.05	0.41	< LOD	0.17	0.14	< LOD	0.08	0.11	< LOD	< LOD
	**April**	4.67	1.87	0.73	2.07	0.05	0.99	0.32	4.03	0.02	0.40	0.65	0.05	0.28	< LOD	0.16	0.10	< LOD	0.15	0.10	< LOD	< LOD
	**May**	5.45	3.43	0.85	2.65	< LOD	1.17	0.04	1.75	0.62	< LOD	0.26	0.28	0.42	< LOD	< LOD	< LOD	< LOD	< LOD	0.23	< LOD	< LOD
	**June**	11.59	12.33	3.90	10.19	0.21	3.53	1.70	7.87	0.10	1.31	1.99	nd	1.21	nd	0.13	0.08	nd	0.06	< LOD	< LOD	< LOD
	**July**	12.30	13.50	4.10	11.40	0.21	4.70	1.20	8.50	0.08	2.30	1.94	nd	2.45	nd	0.17	0.08	nd	< LOD	< LOD	< LOD	< LOD
	**August**	7.26	7.32	4.33	11.00	0.16	6.62	0.86	11.12	0.07	3.46	1.83	nd	2.62	nd	0.25	< LOD	< LOD	< LOD	< LOD	< LOD	< LOD
	**September**	6.44	6.10	1.55	7.44	0.21	4.49	0.89	9.88	0.09	2.97	1.89	0.07	2.54	nd	0.19	< LOD	0.06	< LOD	< LOD	< LOD	< LOD
	**October**	3.89	4.63	3.13	3.62	0.11	2.02	0.86	3.42	0.05	0.56	1.02	< LOD	0.50	< LOD	0.11	< LOD	0.07	< LOD	< LOD	< LOD	< LOD
	**November**	3.77	3.75	2.45	3.26	0.10	1.53	0.71	2.66	0.05	0.53	1.11	nd	0.55	< LOD	0.08	< LOD	< LOD	< LOD	< LOD	< LOD	< LOD
	**December**	3.45	3.39	2.01	2.54	0.12	1.21	0.54	1.87	0.04	0.51	0.97	nd	0.41	< LOD	0.08	< LOD	< LOD	< LOD	< LOD	< LOD	< LOD
	**January**	2.97	3.27	1.37	1.87	0.09	0.90	0.33	1.68	0.04	0.48	0.83	< LOD	0.39	< LOD	0.09	< LOD	< LOD	< LOD	< LOD	< LOD	< LOD
	**February**	1.11	0.81	0.85	0.76	0.07	0.31	0.23	0.77	0.04	0.12	0.48	< LOD	0.13	< LOD	< LOD	< LOD	< LOD	< LOD	< LOD	< LOD	< LOD
	**March**	3.28	1.32	1.03	1.14	0.04	0.52	0.25	0.97	0.02	0.19	0.30	nd	0.18	0.07	< LOD	< LOD	nd	< LOD	< LOD	< LOD	< LOD
GEN	**March**	2.99	4.38	0.68	3.92	0.13	1.40	0.80	2.71	0.04	0.43	1.04	< LOD	0.48	nd	0.11	0.09	< LOD	0.08	0.09	< LOD	< LOD
	**April**	6.11	7.50	1.50	6.50	0.17	3.07	0.72	7.06	0.07	0.92	1.81	< LOD	0.75	< LOD	0.16	< LOD	< LOD	0.06	0.11	< LOD	< LOD
	**May**	10.98	6.52	1.55	5.63	0.02	2.14	0.76	4.03	0.64	0.14	1.11	0.27	0.59	< LOD	< LOD	< LOD	< LOD	< LOD	0.24	< LOD	< LOD
	**June**	14.49	12.33	2.60	12.37	nd	4.07	2.24	10.06	nd	nd	1.51	nd	nd	nd	nd	3.28	nd	nd	nd	nd	nd
	**July**	15.20	12.80	2.90	12.90	0.15	5.30	2.10	11.30	0.30	1.12	1.43	nd	0.82	< LOD	0.07	1.50	nd	nd	nd	nd	nd
	**August**	7.65	7.42	2.39	14.14	0.17	5.98	1.29	7.44	0.09	1.22	0.85	nd	0.77	nd	< LOD	< LOD	< LOD	< LOD	< LOD	< LOD	< LOD
	**September**	6.91	5.18	1.17	7.74	0.18	5.07	1.08	7.24	0.10	2.14	1.48	nd	2.12	nd	0.31	0.15	< LOD	< LOD	0.09	0.23	0.33
	**October**	4.18	9.37	1.48	6.55	0.14	3.38	1.15	5.15	0.10	0.79	0.95	< LOD	0.47	< LOD	0.09	< LOD	0.16	< LOD	< LOD	< LOD	< LOD
	**November**	3.60	7.17	2.74	4.08	0.23	1.98	1.05	3.03	0.09	0.55	0.82	nd	0.47	< LOD	0.09	< LOD	< LOD	< LOD	< LOD	< LOD	< LOD
	**December**	4.02	7.11	2.14	4.32	0.19	2.10	0.78	3.12	0.08	0.67	1.57	nd	0.54	< LOD	0.09	< LOD	< LOD	< LOD	< LOD	< LOD	< LOD
	**January**	4.78	7.01	1.93	4.98	0.10	2.00	0.52	3.11	0.07	0.71	2.13	< LOD	1.03	< LOD	0.15	0.15	< LOD	< LOD	< LOD	< LOD	< LOD
	**February**	1.89	4.64	1.87	2.97	0.13	1.44	0.57	1.96	0.07	0.34	0.76	< LOD	0.29	< LOD	< LOD	< LOD	< LOD	< LOD	< LOD	< LOD	< LOD
	**March**	3.06	4.44	1.29	3.34	0.08	1.41	0.57	2.12	0.04	0.34	0.77	nd	0.35	0.10	< LOD	< LOD	< LOD	< LOD	< LOD	< LOD	< LOD
NES	**March**	1.98	1.67	0.42	1.66	0.07	0.65	0.28	1.62	0.03	0.29	0.61	< LOD	0.26	0.26	0.07	< LOD	0.05	0.05	< LOD	< LOD	< LOD
	**April**	4.49	1.19	0.75	1.67	0.03	1.02	0.24	3.79	0.02	0.42	0.66	< LOD	0.39	< LOD	0.11	0.08	< LOD	0.13	0.08	< LOD	< LOD
	**May**	14.69	7.93	1.38	5.23	< LOD	2.32	0.57	4.87	0.79	0.45	1.29	< LOD	0.86	< LOD	< LOD	< LOD	< LOD	< LOD	0.27	< LOD	< LOD
	**June**	19.18	10.45	2.32	12.38	0.20	6.50	1.07	10.95	0.13	2.16	1.78	nd	1.08	nd	0.11	0.11	nd	0.06	< LOD	< LOD	nd
	**July**	19.80	11.20	2.38	11.10	0.21	5.10	1.30	10.40	0.11	1.96	1.76	nd	1.04	nd	0.08	0.08	nd	0.05	< LOD	< LOD	nd
	**August**	3.92	7.30	2.48	5.96	0.19	2.90	1.26	4.71	0.12	0.92	1.05	0.11	0.59	nd	0.07	< LOD	0.09	< LOD	< LOD	< LOD	< LOD
	**September**	4.64	3.11	8.55	4.34	0.10	1.60	0.32	4.17	0.04	0.64	0.52	nd	0.44	< LOD	< LOD	< LOD	nd	< LOD	< LOD	< LOD	< LOD
	**October**	4.44	4.40	1.46	3.54	0.10	2.04	0.68	2.95	0.09	0.67	0.91	< LOD	0.56	< LOD	0.08	< LOD	< LOD	< LOD	< LOD	< LOD	< LOD
	**November**	4.16	3.90	1.50	3.05	0.11	1.69	0.44	3.61	0.05	0.94	2.22	nd	1.37	< LOD	0.20	0.11	< LOD	0.12	0.07	0.16	< LOD
	**December**	3.56	4.12	0.97	1.59	0.10	0.98	0.35	2.51	0.05	0.54	1.54	nd	0.84	< LOD	< LOD	< LOD	< LOD	< LOD	< LOD	< LOD	< LOD
	**January**	2.08	4.83	0.67	0.97	0.09	0.49	0.15	0.97	0.03	0.34	0.56	< LOD	0.34	< LOD	< LOD	< LOD	< LOD	< LOD	< LOD	< LOD	< LOD
	**February**	1.62	2.37	0.70	1.28	0.14	0.59	0.47	0.91	0.05	0.19	0.62	< LOD	0.16	< LOD	< LOD	< LOD	< LOD	< LOD	< LOD	< LOD	< LOD
	**March**	2.84	1.22	0.64	1.22	0.03	0.38	0.20	0.84	0.03	0.13	0.28	nd	0.13	< LOD	< LOD	< LOD	< LOD	< LOD	< LOD	< LOD	< LOD
SAO	**March**	2.34	0.80	0.94	1.13	0.02	0.29	0.08	1.33	0.01	0.17	0.16	0.05	0.14	< LOD	0.09	< LOD	< LOD	0.07	0.08	< LOD	< LOD
	**April**	1.81	0.88	0.79	1.12	< LOD	0.41	0.05	3.53	0.01	0.22	0.18	< LOD	0.13	< LOD	0.09	< LOD	0.12	0.11	0.11	< LOD	< LOD
	**May**	3.59	1.65	1.71	1.06	< LOD	0.46	< LOD	0.38	0.58	< LOD	< LOD	0.26	< LOD	< LOD	< LOD	< LOD	< LOD	< LOD	0.29	< LOD	< LOD
	**June**	9.65	4.58	4.52	4.81	0.08	1.39	0.34	5.02	0.05	0.74	0.76	nd	0.74	nd	0.14	0.08	nd	0.09	0.05	< LOD	< LOD
	**July**	10.30	5.40	5.30	5.00	0.08	2.00	0.40	5.20	0.04	1.00	1.00	nd	0.98	nd	0.08	0.07	nd	< LOD	0.09	< LOD	< LOD
	**August**	8.61	7.80	13.29	7.94	0.09	2.42	0.48	5.93	0.04	1.05	1.27	nd	1.19	nd	0.09	< LOD	nd	< LOD	< LOD	< LOD	< LOD
	**September**	8.62	4.76	3.85	5.99	0.24	3.94	0.85	7.05	0.09	2.36	2.03	nd	2.90	nd	0.43	0.20	0.12	< LOD	0.06	< LOD	< LOD
	**October**	2.88	2.39	5.72	3.13	0.08	0.90	0.23	2.57	0.05	0.32	0.44	0.05	0.25	0.01	< LOD	< LOD	0.61	< LOD	< LOD	< LOD	< LOD
	**November**	3.66	3.81	5.43	3.41	0.09	0.87	0.24	1.60	0.05	0.19	0.34	nd	0.18	< LOD	< LOD	< LOD	< LOD	0.01	< LOD	< LOD	< LOD
	**December**	2.47	2.10	3.54	1.87	0.06	0.57	0.20	1.21	0.05	0.09	0.24	nd	0.09	< LOD	< LOD	< LOD	< LOD	< LOD	< LOD	< LOD	< LOD
	**January**	1.54	1.30	1.07	0.75	0.05	0.28	0.11	0.63	0.05	0.07	0.12	< LOD	0.07	< LOD	< LOD	< LOD	< LOD	< LOD	< LOD	< LOD	< LOD
	**February**	0.45	0.40	0.97	0.75	0.03	0.29	0.08	0.65	0.03	0.07	0.10	< LOD	0.09	< LOD	< LOD	< LOD	< LOD	< LOD	< LOD	< LOD	< LOD
	**March**	2.45	0.69	1.17	1.02	0.02	0.25	0.10	0.68	0.02	0.08	0.10	nd	0.08	< LOD	< LOD	< LOD	nd	< LOD	< LOD	< LOD	< LOD
TOP	**March**	1.26	0.43	0.17	0.62	nd	0.17	0.06	0.78	0.01	0.10	0.11	< LOD	0.11	0.08	0.07	0.08	< LOD	0.08	0.09	< LOD	< LOD
	**April**	2.24	< LOD	0.34	1.12	< LOD	0.44	0.05	3.25	0.09	0.31	0.45	0.14	0.20	0.09	0.30	0.24	0.06	0.18	0.22	0.29	0.24
	**May**	10.33	4.22	0.28	1.56	< LOD	0.50	< LOD	1.79	0.62	< LOD	0.13	0.25	0.25	< LOD	0.10	< LOD	< LOD	0.06	0.26	< LOD	< LOD
	**June**	8.58	2.25	0.75	2.11	nd	0.56	0.11	3.11	0.04	0.33	0.50	nd	0.44	nd	0.05	< LOD	nd	< LOD	0.06	< LOD	nd
	**July**	9.30	2.24	0.93	2.13	nd	0.64	0.08	2.30	0.04	0.31	0.43	nd	0.34	nd	0.06	< LOD	nd	nd	0.07	< LOD	nd
	**August**	0.94	1.88	1.04	2.58	0.04	0.66	0.08	1.62	0.04	0.32	0.34	nd	0.23	nd	< LOD	< LOD	< LOD	< LOD	< LOD	< LOD	< LOD
	**September**	1.77	1.04	0.88	1.67	0.04	0.53	0.08	1.06	< LOD	0.23	0.24	< LOD	0.23	nd	< LOD	< LOD	< LOD	< LOD	< LOD	< LOD	< LOD
	**October**	1.61	0.61	0.56	1.22	0.04	0.31	0.17	0.82	0.04	0.17	0.27	< LOD	0.17	< LOD	0.09	< LOD	< LOD	< LOD	< LOD	< LOD	< LOD
	**November**	0.53	0.78	0.32	0.53	0.02	0.11	0.07	0.44	0.04	0.06	0.18	nd	0.10	< LOD	< LOD	< LOD	< LOD	0.01	< LOD	< LOD	< LOD
	**December**	0.59	0.54	0.30	0.52	0.02	0.19	0.07	0.32	0.03	0.07	0.21	nd	0.10	< LOD	< LOD	< LOD	< LOD	< LOD	< LOD	< LOD	< LOD
	**January**	0.68	0.36	0.35	0.57	0.03	0.24	0.06	0.34	0.02	0.08	0.20	< LOD	0.11	< LOD	< LOD	< LOD	< LOD	< LOD	< LOD	< LOD	< LOD
	**February**	0.59	0.27	0.20	0.33	0.03	0.08	0.05	0.21	0.02	0.06	0.14	< LOD	0.05	< LOD	< LOD	< LOD	< LOD	< LOD	< LOD	< LOD	< LOD
	**March**	1.52	0.68	0.23	0.52	0.01	0.11	0.05	0.30	0.01	0.07	0.13	nd	< LOD	< LOD	< LOD	< LOD	nd	< LOD	< LOD	< LOD	< LOD
TVN	**March**	4.34	1.49	0.58	2.44	0.05	0.52	0.19	2.27	0.03	0.37	0.48	0.05	0.28	0.05	0.15	0.14	0.05	0.09	0.10	< LOD	< LOD
	**April**	4.03	1.95	0.76	2.51	0.08	0.60	0.14	5.43	0.02	0.40	0.38	0.05	0.23	< LOD	0.11	0.08	< LOD	0.11	0.13	0.21	< LOD
	**May**	6.28	3.33	0.73	2.38	< LOD	0.83	< LOD	1.07	0.59	nd	< LOD	0.26	0.18	< LOD	< LOD	< LOD	< LOD	< LOD	0.26	< LOD	< LOD
	**June**	6.90	5.06	1.33	3.62	0.05	0.79	0.19	3.06	0.03	0.43	0.69	nd	0.40	nd	< LOD	0.08	nd	0.06	< LOD	< LOD	nd
	**July**	7.40	4.86	1.27	4.10	0.05	0.88	0.19	2.98	0.04	0.42	0.66	nd	0.42	nd	< LOD	< LOD	nd	< LOD	< LOD	< LOD	nd
	**August**	1.53	2.93	1.11	4.58	0.04	0.98	0.13	2.38	0.04	0.42	0.51	nd	0.31	nd	< LOD	< LOD	< LOD	< LOD	< LOD	< LOD	< LOD
	**September**	1.79	1.75	1.31	3.14	0.03	0.71	0.15	1.97	0.02	0.46	0.50	nd	0.25	nd	< LOD	0.09	nd	< LOD	< LOD	< LOD	0.21
	**October**	2.99	2.25	1.02	2.79	0.08	0.86	0.22	1.54	0.04	0.28	0.61	< LOD	0.25	< LOD	0.08	< LOD	< LOD	< LOD	< LOD	< LOD	< LOD
	**November**	1.55	2.10	0.85	1.67	0.06	0.53	0.18	1.06	0.04	0.20	0.62	nd	0.22	< LOD	0.06	< LOD	< LOD	0.05	0.08	0.24	< LOD
	**December**	1.42	1.87	0.78	1.08	0.06	0.35	0.10	0.78	0.03	0.19	0.38	nd	0.10	< LOD	< LOD	< LOD	< LOD	< LOD	< LOD	< LOD	< LOD
	**January**	1.51	1.26	0.59	0.92	0.06	0.25	0.08	0.57	0.05	0.12	0.29	< LOD	0.11	< LOD	< LOD	< LOD	< LOD	< LOD	< LOD	< LOD	< LOD
	**February**	1.49	1.63	0.53	1.01	0.03	0.23	0.10	0.61	0.02	0.12	0.30	< LOD	0.10	< LOD	< LOD	< LOD	< LOD	< LOD	< LOD	< LOD	< LOD
	**March**	3.40	0.63	0.51	1.20	0.02	0.34	0.10	0.77	0.05	0.15	0.44	nd	0.16	0.07	< LOD	< LOD	nd	< LOD	< LOD	< LOD	< LOD

### Statistical analysis

**Fig. 3:**
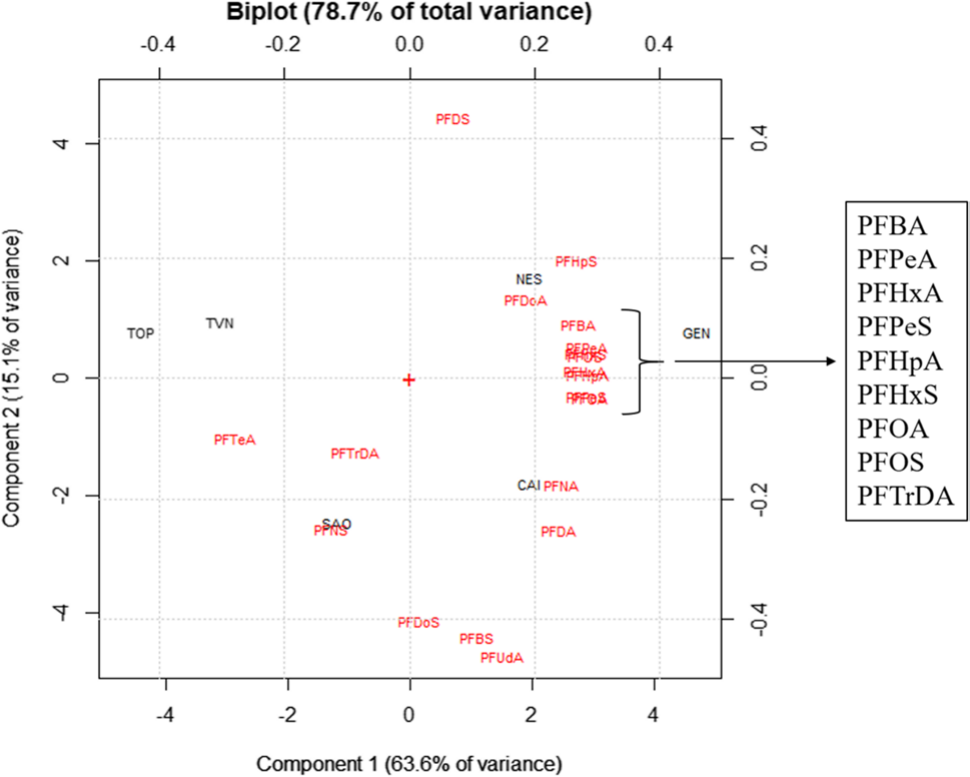
Biplot (PC1 and PC2) of the PCA performed on the concentration data of twenty-one PFASs detected in six rivers (TOP, TVN, SAO, CAI, NES, and GEN) of central Italy

PCA results are reported in the biplot in Fig. 3, while scores and loadings are summarized in Tables S2 and S3 (supplementary material), respectively. The two obtained significant components (PC1 and PC2) explained 78.7% of the total variance. Component 1 (63.6% of the total variance; Fig. 3) well separated two clusters of river waters, each characterized by its contamination profile. In the left part of the biplot, the first cluster is characterized by three rivers (TOP, TVN, and SAO) and three PFASs (PFTeA, PFNS, and PFTrDA). All three rivers previously mentioned were impacted by several industries (paper mills, cement plants, and other smaller industrial activities) that discharged their wastewater directly into the rivers. In this case, these three long-chain PFASs (PFTeA, PFNS, and PFTrDA) can be considered tracers of the previously mentioned industrial activities. Chow and Foo (2023) analyzed PFASs in paper mill wastewaters finding a contamination profile characterized by PFOA, PFOS, and PFHxA. Hale et al. (2021) measured PFASs in six different sites of lake Tyrifjorden (Norway), highly impacted by a factory producing paper products, finding a contamination profile characterized by PFOS, PFHxA, and PFPeA. Also, Langberg et al. (2021) determined the chemical composition of wastewater produced by a paper mill in Norway, finding a profile dominated by PFOA, PFOS, and, as a smaller proportion, by C5-C7 and C9 perfluorinated carboxylic acids. All three previously cited works reported a contamination profile quite different from one another and different from that reported in this study. This could be explained considering the different types of treated raw materials. Unfortunately, to our knowledge, no study investigated the release of PFASs from cement plants; for this reason, a comparison with the literature is difficult. In the right part of the biplot, the second cluster consists of three rivers (GEN, NES, and CAI) and several PFASs (Fig. 3). The three rivers composing the second cluster were affected by both urban wastewater and livestock holding discharges. Kolpin et al. (2021) analyzed water samples collected in rivers strongly impacted by urban and agricultural activities and livestock production, finding a contamination profile dominated by PFBS, PFOA, PFHxA, and PFPeA. Also, Tuan et al. (2021) found high concentrations of PFBA, PFPeA, PFHxS, and PFHxA in river water samples affected by agricultural production, livestock farming, and urban wastewater discharges.

### Ecological risk assessment

**Fig. 4:**
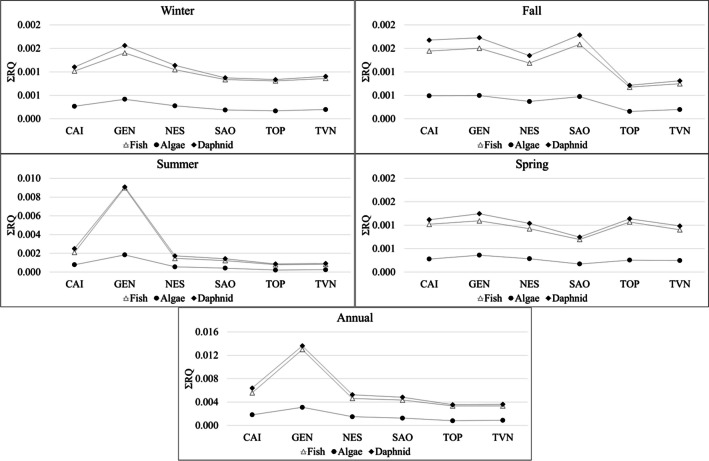
Total risk quotients (ΣRQs) calculated seasonally and annually for three aquatic model organisms (algae, daphnids, and fish) in six different rivers from Umbria region (central Italy)

The total risk quotients (ΣRQs) for three typical organisms (fish, algae, and daphnid) calculated seasonally (winter, fall, summer, and spring) and annually (March 2022–March 2023) for the six rivers under study are reported in Fig. 4. The ΣRQs were higher in GEN river for the three aquatic organisms in winter, summer, and spring (Fig. 4). During fall season, instead, the ΣRQs were higher in SAO river for all the aquatic organisms (Fig. 4). During summer, the ΣRQ values for fish and daphnid in GEN river significantly increased, reaching values of 8.99 × 10^−3^ and 9.08 × 10^−3^, respectively (Fig. 4). However, the risk for fish, algae, and daphnid during the four seasons was always negligible, with ∑RQ values much lower than 0.01 (Fig. 4).

Considering the annual exposure, the highest ∑RQs were obtained for GEN river, followed by CAI and NES rivers (Fig. 4). In detail, the risk for fish (values between 3.34 × 10^−3^ and 1.03 × 10^−2^; Fig. 4) was negligible in all the rivers under study, except for GEN river (low risk). For the daphnid, the risk was low in GEN river and negligible in all the other rivers (∑RQs between 3.55 × 10^−3^ and 1.36 × 10^−2^; Fig. 4). For the algae, the risk was always negligible, with ∑RQ values between 8.04 × 10^−4^ and 3.11 × 10^−3^ (Fig. 4).

## Conclusions

The contamination data obtained from 1638 analytical determinations of twenty-one PFASs in six different rivers of Umbria region (central Italy) during a 13-month monitoring campaign showed a situation of quite low contamination. The majority of the detected PFASs were consistent with the EQS established by Italian 172/2015 Decree Law. The single exception was PFOS: the concentrations of this compound exceeded the EQS in 47% of the analyzed samples. The monthly trend of the Σ_21_PFCS was characterized by higher concentrations in the hottest months (from June to September) in all the investigated rivers, probably due to a reduced river flow caused by high temperatures and low rainfall. The ecological risk assessment, based on the calculation of monthly and annual ∑RQs for three aquatic organisms (fish, algae, and daphnid), showed that the risk for fish, algae, and daphnid during the four seasons was always negligible. For the annual exposure, the risk for fish and daphnid was negligible, with the exception of GEN river (low risk). During 1-year monitoring campaign, the risk for the algae was negligible in all the rivers under study. The results obtained in this study confirmed the widespread distribution of PFASs in all the rivers under study, even in an area (central Italy) devoid of direct emission sources. The results of this study could be significant for developing a database to estimate the background contamination of river waters in central Italy.

## Supplementary Information

Below is the link to the electronic supplementary material.

Supplementary file1 (DOCX 19 KB)

## Data Availability

Data available upon request to the corresponding author.

## References

[CR1] Barreca S, Busetto M, Colzani L, Clerici L, Marchesi V, Tremolada L, Daverio D, Dellavedova P (2020) Hyphenated high performance liquid chromatography–tandem mass spectrometry techniques for the determination of perfluorinated alkylated substances in Lombardia Region in Italy, profile levels and assessment: one year of monitoring activities during 2018. Separations 7:17. 10.3390/separations7010017

[CR2] Cai Y, Wang X, Wu Y, Zhao S, Li Y, Ma L, Chen C, Huang J, Yuet G (2018) Temporal trends and transport of perfluoroalkyl substances (PFASs) in a subtropical estuary: Jiulong River Estuary, Fujian, China. Sci Total Environ 639:263–270. 10.1016/j.scitotenv.2018.05.042 29787910 10.1016/j.scitotenv.2018.05.042

[CR3] Castellani F, Galletti M, Charavgis F, Cingolani A, Renzi S, Nucci M, Protano C, Vitali M (2023) Perfluorinated compounds (PFCs) in river waters of Central Italy: monthly variation and ecological risk assessment (ERA). Arch Environ Contam Toxicol 84:332–346. 10.1007/s00244-023-00993-4 37022436 10.1007/s00244-023-00993-4 PMC10130131

[CR4] Castiglioni S, Valsecchi S, Polesello S, Rusconi M, Melis M, Palmiotto M, Manenti A, Davoli E, Zuccato E (2015) Sources and fate of perfluorinated compounds in the aqueous environment and in drinking water of a highly urbanized and industrialized area in Italy. J Hazard Mater 282:51–60. 10.1016/j.jhazmat.2014.06.007 24986164 10.1016/j.jhazmat.2014.06.007

[CR5] Charavgis F, Cingolani A, Renzi S (2022) Il monitoraggio delle sostanze perfluoroalchiliche nelle acque superficiali e sotterranee della regione Umbria (2018–2021). ARPA Umbria

[CR6] Chen R, Li G, Yu Y, Ma X, Zhuang Y, Tao H, Shi B (2019) Occurrence and transport behaviors of perfluoroalkyl acids in drinking water distribution systems. Sci Total Environ 697:134162. 10.1016/j.scitotenv.2019.134162 31491637 10.1016/j.scitotenv.2019.134162

[CR7] Chow YN, Foo KY (2023) Insights into the per-and polyfluoroalkyl substances-contaminated paper mill processing discharge: detection, phytotoxicity, bioaccumulative profiling, and health risk verification. J Clean Prod 384:135478. 10.1016/j.jclepro.2022.135478

[CR8] Dasu K, Xia X, Siriwardena D, Klupinski TP, Seay B (2022) Concentration profiles of per-and polyfluoroalkyl substances in major sources to the environment. J Environ Manag 301:113879. 10.1016/j.jenvman.2021.113879 10.1016/j.jenvman.2021.113879 34619593

[CR9] EPA (Environmental Protection Agency) Method 1633 (2022). Analysis of per- and polyfluoroalkyl substances (PFAS) in aqueous, solid, biosolids, and tissue samples by LC-MS/MS

[CR10] European Commission (EC) (2013) Directive 2013/39/EU of the European parliament and of the council of 12 August 2013 amending Directives 2000/60/EC and 2008/105/EC as regards priority substances in the field of water policy. Official Journal of the European Union. L 226/1

[CR11] Giglioli S, Colombo L, Azzellino A (2023) Cluster and multivariate analysis to study the diffuse contamination of emerging per-and polyfluoroalkyl substances (PFAS) in the Veneto Region plain (North-eastern Italy). Chemosphere 319:137916. 10.1016/j.chemosphere.2023.137916 36706810 10.1016/j.chemosphere.2023.137916

[CR12] Hale SE, Canivet B, Rundberget T, Langberg HA, Allan IJ (2021) Using passive samplers to track per and polyfluoroalkyl substances (PFAS) emissions from the paper industry: laboratory calibration and field verification. Front Environ Sci 9:621. 10.3389/fenvs.2021.796026

[CR13] Kolpin DW, Hubbard LE, Cwiertny DM, Meppelink SM, Thompson DA, Gray JL (2021) A comprehensive statewide spatiotemporal stream assessment of per-and polyfluoroalkyl substances (PFAS) in an agricultural region of the United States. Environ Sci Technol Lett 8:981–988. 10.1021/acs.estlett.1c00750

[CR14] Langberg HA, Arp HPH, Breedveld GD, Slinde GA, Høiseter Å, Grønning HM, Jartun M, Rundberget T, Jenssen BM, Hale SE (2021) Paper product production identified as the main source of per-and polyfluoroalkyl substances (PFAS) in a Norwegian lake: source and historic emission tracking. Environ Pollut 273:116259. 10.1016/j.envpol.2020.116259 10.1016/j.envpol.2020.116259 33450507

[CR15] Lee JW, Choi K, Park K, Seong C, Yu SD, Kim P (2020) Adverse effects of perfluoroalkyl acids on fish and other aquatic organisms: a review. Sci Total Environ 707:135334. 10.1016/j.scitotenv.2019.135334 31874399 10.1016/j.scitotenv.2019.135334

[CR16] Leng Y, Xiao H, Li Z, Liu Y, Huang K, Wang J (2021) Occurrence and ecotoxicological risk assessment of perfluoroalkyl substances in water of lakes along the middle reach of Yangtze River. China Sci Total Environ 788:147765. 10.1016/j.scitotenv.2021.147765 34022575 10.1016/j.scitotenv.2021.147765

[CR17] Li J, Ai Y, Hu J, Xu N, Song R, Zhu Y, Sun W, Ni J (2020a) Polyfluoroalkyl substances in Danjiangkou reservoir, China: occurrence, composition, and source appointment. Sci Total Environ 725:138352. 10.1016/j.scitotenv.2020.138352 32278931 10.1016/j.scitotenv.2020.138352

[CR18] Li J, Gao Y, Xu N, Li B, An R, Sun W, Borthwick AGL, Ni J (2020b) Perfluoroalkyl substances in the Yangtze River: changing exposure and its implications after operation of the Three Gorges Dam. Water Res 182:115933. 10.1016/j.watres.2020.115933 32650148 10.1016/j.watres.2020.115933

[CR19] Liu X, Zheng X, Zhang L, Li J, Li Y, Huang H, Fan Z (2022) Joint toxicity mechanisms of binary emerging PFAS mixture on algae (Chlorella pyrenoidosa) at environmental concentration. J Hazard Mater 437:129355. 10.1016/j.jhazmat.2022.129355 35716567 10.1016/j.jhazmat.2022.129355

[CR20] Llorens E, Ginebreda A, la Farré M, Insa S, González-Trujillo JD, Munné A, Solà C, Flò M, Villagrasa M, Barceló D, Sabater S (2020) Occurrence of regulated pollutants in populated Mediterranean basins: ecotoxicological risk and effects on biological quality. Sci Total Environ 747:141224. 10.1016/j.scitotenv.2020.141224 32771786 10.1016/j.scitotenv.2020.141224

[CR21] Mahoney H, Xie Y, Brinkmann M, Giesy JP (2022) Next generation per-and poly-fluoroalkyl substances: status and trends, aquatic toxicity, and risk assessment. Eco-Environ Health 1(2):117–131. 10.1016/j.eehl.2022.05.002 38075527 10.1016/j.eehl.2022.05.002 PMC10702929

[CR22] Munoz G, Budzinski H, Babut M, Lobry J, Selleslagh J, Tapie N, Labadie P (2019) Temporal variations of perfluoroalkyl substances partitioning between surface water, suspended sediment, and biota in a macrotidal estuary. Chemosphere 233:319–326. 10.1016/j.chemosphere.2019.05.281 31176133 10.1016/j.chemosphere.2019.05.281

[CR23] Nayak S, Sahoo G, Das II, Mohanty AK, Kumar R, Sahoo L, Sundaray JK (2023) Poly- and perfluoroalkyl substances (PFAS): do they matter to aquatic ecosystems? Toxics 11:543. 10.3390/toxics11060543 37368643 10.3390/toxics11060543 PMC10302656

[CR24] Nucci M, Cingolani A, Charavgis F, Renzi S (2019a) Le sostanze perfluoroalchiliche (PFAS) in Umbria: stato attuale e programmi di monitoraggio. ARPA Umbria, Micron online

[CR25] Nucci M, Cingolani A, Charavgis F, Renzi S (2019b) I PFAS in Umbria nel 2018 - monitoraggio delle sostanze perfluoroalchiliche su acque superficiali, acque sotterranee e scarichi in Umbria. ARPA Umbria, Micron online

[CR26] Organization for Economic Co-operation and Development (2018) Toward a new comprehensive global database of per- and polyfluoroalkyl substances (PFASs): summary report on updating the OECD 2007 List of Per- and Polyfluoroalkyl SubstancES (PFASs)

[CR27] Saawarn B, Mahanty B, Hait S, Hussain S (2022) Sources, occurrence, and treatment techniques of per-and polyfluoroalkyl substances in aqueous matrices: a comprehensive review. Environ Res 214:114004. 10.1016/j.envres.2022.114004 35970375 10.1016/j.envres.2022.114004

[CR28] Selvaraj KK, Murugasamy M, Nikhil NP, Elaiyaraja A, Sampath S, Krishnamoorthi V, He H, Ramaswamy BR (2021) Investigation of distribution, sources and flux of perfluorinated compounds in major southern Indian rivers and their risk assessment. Chemosphere 277:130228. 10.1016/j.chemosphere.2021.130228 34384168 10.1016/j.chemosphere.2021.130228

[CR29] Tuan DH, Anh PTL, Lam BN (2021) Distribution of perfluoroalkyl substances (PFASs) in the water of the Bac Hung Hai River, Van Giang district, Hung Yen province, Vietnam. VN J Hydrometeorol 9:46–53. 10.36335/VNJHM

[CR30] UNEP (United Nations Environment Program) (2009) Decision SC-4/17: listing of PFOS, its salts and perfluorooctane sulfonyl fluoride (PFOSF) in Annex B to the Stockholm Convention. UN Environment (UNEP), Secretariat of the Basel, Rotterdam and Stockholm Conventions, Geneva, Switzerland

[CR31] UNEP (United Nations Environment Program) (2019) Decision SC-9/12: listing of Perfluorooctanoic acid (2019), its salts and PFOA-related compounds. UN Environment (UNEP), Secretariat of the Basel, Rotterdam and Stockholm Conventions, Geneva, Switzerland

[CR32] UNEP (United Nations Environment Program) (2022a) Decision SC-10/13: listing of perfluorohexane sulfonic acid (PFHxS), its salts and PFHxS-related compounds. UN Environment (UNEP), Secretariat of the Basel, Rotterdam and Stockholm Conventions, Geneva, Switzerland

[CR33] UNEP (United Nations Environment Program) (2022b) Proposal to list long-chain perfluorocarboxylic acids, their salts and related compounds in Annexes A, B and/or C to the Stockholm Convention on Persistent Organic Pollutants. UNEP/POPS/POPRC.17/7

[CR34] US EPA (United States Environmental Protection Agency) Method 533 (2019) Method 533: determination of per- and polyfuoroalkyl substances in drinking water by isotope dilution anion exchange solid phase extraction and liquid chromatography/tandem mass spectrometry. https://www.epa.gov/sites/default/files/2019-12/documents/method-533-815b19020.pdf. Accessed 20 Nov 2023

[CR35] Valsecchi S, Rusconi M, Mazzoni M, Viviano G, Pagnotta R, Zaghi C, Serrini G, Polesello S (2015) Occurrence and sources of perfluoroalkyl acids in Italian river basins. Chemosphere 129:126–134. 10.1016/j.chemosphere.2014.07.044 25108894 10.1016/j.chemosphere.2014.07.044

[CR36] Wang S, Cai Y, Ma L, Lin X, Li Q, Li Y, Wang X (2022) Perfluoroalkyl substances in water, sediment, and fish from a subtropical river of China: environmental behaviors and potential risk. Chemosphere 288:132513. 10.1016/j.chemosphere.2021.132513 34634273 10.1016/j.chemosphere.2021.132513

[CR37] Wu J, Junaid M, Wang Z, Sun W, Xu N (2020) Spatiotemporal distribution, sources and ecological risks of perfluorinated compounds (PFCs) in the Guanlan River from the rapidly urbanizing areas of Shenzhen. China Chemosphere 245:125637. 10.1016/j.chemosphere.2019.125637 31864951 10.1016/j.chemosphere.2019.125637

[CR38] Yao Y, Burgess J, Volchek K, Brown CE (2018) Short-chain PFAS: their sources, properties, toxicity, environmental fate, and treatment. In Perfluoroalkyl Substances in the Environment. 447–466 CRC Press

[CR39] Zafeiraki E, Gebbink WA, Hoogenboom RLAP, Kotterman M, Kwadijk C, Dassenakis E, van Leeuwen SPJ (2019) Occurrence of perfluoroalkyl substances (PFASs) in a large number of wild and farmed aquatic animals collected in the Netherlands. Chemosphere 232:415–423. 10.1016/j.chemosphere.2019.05.200 31158636 10.1016/j.chemosphere.2019.05.200

[CR40] Zhang F, Wang Y, Wei Z, Zhang G, Wang J (2021a) Perfluorinated compounds in a river basin from QingHai-Tibet Plateau: occurrence, sources and key factors. Ecotoxicol Environ Saf 228:113043. 10.1016/j.ecoenv.2021.113043 34863078 10.1016/j.ecoenv.2021.113043

[CR41] Zhang Y, Zhou Y, Zhang A, Li J, Yu J, Dou Y, He J, Kong D (2021b) Perfluoroalkyl substances in drinking water sources along the Yangtze River in Jiangsu Province, China: human health and ecological risk assessment. Ecotoxicol Environ Saf 218:112289. 10.1016/j.ecoenv.2021.112289 33940442 10.1016/j.ecoenv.2021.112289

[CR42] Zhang K, Sumita LC, Sun C, Marmier N (2022) A review of the treatment process of perfluorooctane compounds in the waters: adsorption, flocculation, and advanced oxidative process. Water 14:2692. 10.3390/w14172692

[CR43] Zhou Y, Zhou Z, Lian Y, Sun X, Wu Y, Qiao L, Wang M (2021) Source, transportation, bioaccumulation, distribution and food risk assessment of perfluorinated alkyl substances in vegetables: a review. Food Chem 349:129137. 10.1016/j.foodchem.2021.129137 33556727 10.1016/j.foodchem.2021.129137

[CR44] Zhu Z, Wang T, Meng J, Wang P, Li Q, Lu Y (2015) Perfluoroalkyl substances in the Daling River with concentrated fluorine industries in China: seasonal variation, mass flow, and risk assessment. Environ Sci Pollut Res 22:10009–10018. 10.1007/s11356-015-4189-0 10.1007/s11356-015-4189-0 25666478

