# Supplementary material for: Perfluoroalkyl substances: a risk for the aquatifc environment? A 1-year case study in river waters of central Italy
Source: Environ Sci Pollut Res Int. 2024 Sep 18;32(16):10464–75. doi: 10.1007/s11356-024-34807-4 (PMC11996964; doi:10.1007/s11356-024-34807-4)
Supplement: Supplementary file 1 — Supplementary file1 (DOCX 19 KB) [file 11356_2024_34807_MOESM1_ESM.docx]

**Perfluoroalkyl substances: a risk for the aquatic environment? A one-year case-study in river waters of Central Italy**

Federica Castellani^a^, Mara Galletti^b^, Fedra Charavgis^b^, Alessandra Cingolani^b^, Sonia Renzi^b^, Mirko Nucci^b^, Carmela Protano^a^, Matteo Vitali^a,*^

^a^ Department of Public Health and Infectious Diseases, University of Rome La Sapienza, P.le Aldo Moro, 5, 00185 Rome, Italy

^b^ ARPA Umbria, Via Carlo Alberto dalla Chiesa, 23, 05100 Terni, Italy

^c^ ARPA Umbria, Via Pievaiola 207/B-3, 06132 Perugia, Italy

* corresponding Author: matteo.vitali@uniroma1.it

**Table of contents**

**Table S1**

**Table S2**

**Table S3**

**Table S1.** Full names, acronym, retention times (RT; minutes), precursor and product ions, fragmentor (V) and collision energy (eV) of the mass labelled extraction and injection standard standards.

|  | **Full name** | **Acronym** | **RT (min)** | **Precursor Ion (m/z)** | **Product Ion (m/z)** | **Fragmentor (V)** | **Collision Energy (eV)** |
| --- | --- | --- | --- | --- | --- | --- | --- |
| Mass-labelled extraction standards (ES) | Perfluoro-n-(^13^C_4_)butanoic Acid | **MPFBA** | 0.89 | 217 | 172 | 60 | 8 |
|  | Perfluoro-n-(^13^C_5_)pentanoic Acid | **M5PFPeA** | 1.85 | 268 | 223 | 60 | 6 |
|  | Sodium Perfluoro-1-(2,3,4-^13^C_3_)butanesulfonate | **M3PFBS** | 2.13 | 302 | 80 | 133 | 45 |
|  | Perfluoro-n-(1,2,3,4,6-^13^C_5_)hexanoic Acid | **M5PFHxA** | 3.43 | 318 | 273 | 66 | 5 |
|  | Perfluoro-n-(1,2,3,4-^13^C_4_)heptanoic Acid | **M4PFHpA** | 4.83 | 367 | 322 | 66 | 5 |
|  | Sodium Perfluoro-1-(1,2,3-^13^C_3_)hexanesulfonate | **M3PFHxS** | 4.96 | 402 | 80 | 174 | 49 |
|  | Perfluoro-n-(^13^C_8_)octanoic Acid | **M8PFOA** | 5.96 | 421 | 376 | 86 | 5 |
|  | Perfluoro-n-(^13^C_9_)nonanoic Acid | **M9PFNA** | 6.87 | 472 | 427 | 66 | 5 |
|  | Sodium perfluoro-1-(^13^C_8_)octanesulfonate | **M8PFOS** | 6.90 | 507 | 80 | 210 | 50 |
|  | Perfluoro-N-(1,2,3,4,5,6-^13^C_6_)decanoic Acid | **M6PFDA** | 7.64 | 518.9 | 473.9 | 102 | 5 |
|  | Perfluoro-n-(1,2,3,4,5,6,7-^13^C_7_)undecanoic Acid | **M7PFUdA** | 8.30 | 570 | 525 | 92 | 5 |
|  | Perfluoro-n-(1,2-^13^C_2_)dodecanoic Acid | **MPFDoA** | 8.86 | 614.9 | 570 | 97 | 5 |
|  | Perfluoro-n-(1,2-^13^C_2_)tetradecanoic Acid | **M2PFTeDA** | 9.92 | 715 | 670 | 112 | 5 |
| Mass-labelled injection standards (IS) | Perfluoro-n-(2,3,4-^13^C_3_) butanoic Acid | **M3PFBA** | 0.90 | 216 | 172 | 60 | 8 |
|  | Perfluoro-n-(1,2-^13^C_2_) octanoic Acid | **M2PFOA** | 5.96 | 415 | 370 | 86 | 5 |
|  | Sodium Perfluoro-1-(1,2,3,4-^13^C_4_) Octane Sulfonate | **MPFOS** | 6.90 | 503 | 99 | 210 | 50 |
|  | Perfluoro-n-(1,2-^13^C_2_) Decanoic Acid | **MPFDA** | 7.65 | 515 | 470 | 102 | 5 |

**Table S2.** Explained variance (%) and scores of the five components obtained by performing PCA.

|  | **PC1** | **PC2** | **PC3** | **PC4** | **PC5** |
| --- | --- | --- | --- | --- | --- |
| **Variance %** | 63.5808 | 15.0794 | 11.3659 | 8.4999 | 1.4741 |
| **CAI** | 1.9792 | -1.7975 | 1.2269 | -1.8056 | -0.0056 |
| **GEN** | 4.7239 | 0.7870 | -2.1278 | -0.0839 | -0.0266 |
| **NES** | 1.9660 | 1.7157 | 2.0246 | 1.0949 | 0.2228 |
| **SAO** | -1.1769 | -2.4487 | -0.3982 | 1.7445 | 0.0190 |
| **TOP** | -4.3989 | 0.7846 | -0.7120 | -0.7143 | 0.7100 |
| **TVN** | -3.0933 | 0.9590 | -0.0135 | -0.2355 | -0.9195 |

**Table S3.** Loadings of the five components obtained by performing PCA.

|  | **PFBA** | **PFPeA** | **PFBS** | **PFHxA** | **PFPeS** | **PFHpA** | **PFHxS** | **PFOA** | **PFHpS** | **PFNA** | **PFOS** | **PFNS** | **PFDA** | **PFDS** | **PFUdA** | **PFDoA** | **PFDoS** | **PFTrDA** | **PFTeA** |
| --- | --- | --- | --- | --- | --- | --- | --- | --- | --- | --- | --- | --- | --- | --- | --- | --- | --- | --- | --- |
| **PC1** | 0.2714 | 0.2839 | 0.1078 | 0.2806 | 0.2844 | 0.2864 | 0.2808 | 0.2869 | 0.2670 | 0.2446 | 0.2825 | -0.1246 | 0.2388 | 0.0694 | 0.1491 | 0.1881 | 0.0158 | -0.0863 | -0.2787 |
| **PC2** | 0.0892 | 0.0519 | -0.4295 | 0.0116 | -0.0286 | 0.0047 | 0.0421 | -0.0327 | 0.1938 | -0.1755 | 0.0373 | -0.2498 | -0.2522 | 0.4344 | -0.4605 | 0.1322 | -0.4026 | -0.1226 | -0.0991 |
| **PC3** | 0.1192 | -0.0706 | 0.0407 | -0.1213 | 0.0749 | -0.0285 | -0.1121 | 0.0164 | -0.0356 | 0.2611 | 0.0968 | -0.2158 | 0.2085 | 0.3552 | 0.0235 | -0.4913 | -0.1662 | 0.6164 | 0.0263 |
| **PC4** | 0.1705 | -0.0376 | 0.4502 | -0.0441 | -0.0216 | -0.0649 | -0.0959 | -0.0339 | 0.0741 | -0.1596 | -0.0836 | -0.5618 | -0.1395 | 0.1420 | -0.2387 | -0.0267 | 0.5357 | -0.1053 | -0.0323 |
| **PC5** | 0.1854 | -0.1409 | -0.0211 | -0.2175 | -0.1653 | 0.0470 | 0.0445 | -0.0262 | 0.2640 | 0.0363 | -0.0462 | 0.2899 | 0.1097 | 0.5865 | 0.3290 | -0.0149 | 0.1836 | -0.3179 | 0.3307 |
